# Editorial: Deep learning in brain-computer interfaces

**DOI:** 10.3389/fnhum.2026.1888360

**Published:** 2026-06-10

**Authors:** Jordi Solé-Casals, Sangtae Ahn, Bin He

**Affiliations:** 1 Data and Signal Processing Group, University of Vic-Central University of Catalonia, Vic/Barcelona, Spain; 2 Department of Psychiatry, University of Cambridge, Cambridge, United Kingdom; 3 School of Electronic and Electrical Engineering, Kyungpook National University, Daegu, Republic of Korea; 4 Department of Biomedical Engineering, Carnegie Mellon University, Pittsburgh, PA, United States

**Keywords:** brain-computer interfaces (BCI), deep learning—artificial intelligence, EEG decoding, emotion recognition, graph neural networks (GNN), inner speech decoding, motor imagery

Brain-computer interfaces (BCI) are becoming an important meeting point between neuroscience, artificial intelligence, and human-machine interaction. Their main goal is simple to state but difficult to achieve: to use brain signals to support communication, control, rehabilitation, or a better understanding of human mental states. In recent years, deep learning has become one of the main tools driving this field forward, as it can learn complex spatial, spectral, and temporal patterns directly from neural data.

This Research Topic, *Deep learning in brain-computer interfaces*, brings together four articles that illustrate how this area is developing. The contributions span motor imagery decoding, emotion recognition, and inner speech decoding. Despite their different applications, they all face the same underlying difficulty: brain signals are noisy, variable across people, and often limited in size. Progress in BCI, therefore, does not hinge solely on building larger neural networks, it also depends on designing models that respect the structure of brain data and can hold up under realistic conditions.

A central theme of the Research Topic is motor imagery BCI. Motor imagery is widely used because it allows a person to generate commands by imagining movements, without using muscles. EEG-based motor imagery decoding remains difficult, though, especially when several movement classes must be distinguished. In their article, Zhao et al. propose MCFANet, a multi-class fusion attention network for motor imagery EEG classification. The model combines traditional signal processing ideas with deep learning: it uses the spatial filtering capacity of FBCSP while preserving temporal information for convolutional feature extraction. This is a meaningful design choice, showing that classical BCI methods and modern deep learning are not competing alternatives, they can complement each other in ways that retain neurophysiological information while improving the learning capacity of the model.

The second motor imagery contribution, by Shao et al., takes the field further toward network-based modeling. Their DSSICNN framework treats motor imagery EEG as a dynamic spectral-spatial process, including spatial representations in both Euclidean and non-Euclidean domains, modeling cross-frequency interactions, and using attention to capture the varying contributions of different motor imagery stages. EEG activity during motor imagery is not static, it evolves over time and reflects interactions between brain areas and frequency bands. By connecting graph-based deep learning with ideas from network neuroscience, this work offers both a practical decoding method and a richer way of thinking about the neural mechanisms underlying motor imagery.

The Research Topic also includes work beyond motor control. Rahman et al. address EEG-based emotion recognition using Multilayer-GTCN, a graph transformer convolutional network. Their model combines physical proximity and functional connectivity to represent EEG data through complementary graph structures. In affective BCI, the goal is not to decode a movement intention but to identify emotional states from brain activity, and this article shows how graph neural networks (GNN) and transformer-based layers can be used together to capture both local and global relations in EEG signals. More broadly, it points to an expansion of BCI research toward cognitive and affective states, well beyond the traditional focus on control commands.

The mini review by Sümer-Arpak et al. focuses on inner speech decoding from non-invasive brain signals, a particularly promising direction for communication BCIs, especially for people with severe speech impairments or conditions that limit overt communication. The review discusses the possible role of foundation models, including self-supervised approaches, in learning more transferable representations from neural recordings. Given that many BCI datasets are small and heterogeneous, this is a timely direction to explore. At the same time, the review does not shy away from the challenges that remain: signal quality, non-stationarity, lack of standardization, limited interpretability, and ethical concerns related to privacy and imagined language.

Taken together, the articles in this Research Topic reflect a field that is rapidly developing. Deep learning is no longer applied as a black-box classifier on top of pre-processed data. The approaches collected here incorporate spatial filters, attention mechanisms, graph structures, temporal dynamics, spectral interactions, and representation learning. All these design choices bring the models closer to the actual properties of brain signals.

However, important challenges remain. Deep learning models still need to generalize better across subjects, sessions, and recording conditions, and they need to be evaluated more rigorously before they can be deployed outside research laboratory settings. Interpretability is a pressing concern, especially for clinical, rehabilitation, and assistive applications, where understanding why a system works, and when it might fail, is not optional. As BCIs expand toward communication, emotion recognition, and cognitive-state decoding, questions about consent, privacy, and responsible use will only become more pressing. The work collected here makes clear that future progress will depend on a genuine integration of machine learning, signal processing, neuroscience, and real user needs, rather than on advancing each of these in isolation.

